# Sonoelectrochemical Degradation of Propyl Paraben: An Examination of the Synergy in Different Water Matrices

**DOI:** 10.3390/ijerph17082621

**Published:** 2020-04-11

**Authors:** Zacharias Frontistis

**Affiliations:** Department of Chemical Engineering, University of Western Macedonia, GR-50100 Kozani, Greece; zfrontistis@uowm.gr

**Keywords:** electrochemical oxidation, ultrasound, synergy, parabens, water matrices

## Abstract

The synergistic action of anodic oxidation using boron-doped diamond and low-frequency ultrasound in different water matrices and operating conditions for the decomposition of the emerging contaminant propyl paraben was investigated. The degree of synergy was found to decrease with an increase in current in the range 1.25–6.25 mA/cm^2^ or the ultrasound power until 36 W/L, where a further decrease was observed. Despite the fact that the increased propyl paraben concentration decreased the observed kinetic constant for both the separated and the hybrid process, the degree of synergy was increased from 37.3 to 43.4% for 0.5 and 2 mg/L propyl paraben, respectively. Bicarbonates (100–250 mg/L) or humic acid (10–20 mg/L) enhanced the synergy significantly by up to 55.8%, due to the higher demand for reactive oxygen species. The presence of chloride ions decreased the observed synergistic action in comparison with ultrapure water, possibly due to the electro-generation of active chlorine that diffuses to the bulk solution. The same behavior was observed with the secondary effluent that contained almost 68 mg/L of chlorides. The efficiency was favored in a neutral medium, while the hybrid process was delayed in alkaline conditions.

## 1. Introduction

Nowadays, the interest of both the public and the scientific community about the harmful effects of various chemicals called endocrine disruptors (EDCs) has increased [1,2]. These substances can mimic or render the hormones and are accused of affecting the endocrine system [3]. Parabens are among the most well-known endocrine disruptors. Their primary use is as preservatives [4]. Parabens are resistant to biological degradation. Therefore, they have been quantified in a large number of water bodies like rivers and lakes and effluents from wastewater treatment plants worldwide [5,6,7]. In a recent work published by Honda et al. [8] that was performed in 8 different countries, including Asian countries as well as Greece and the USA, the researchers detected propyl paraben (PP) in 80.3% of the urinary samples, with an average concentration of 1.21 μg/L.

For the further treatment of non-biodegradable wastewater, advanced oxidation processes (AOPs) are an interesting alternative. AOPs are a group of physico-chemical technologies that rely on the in situ generation of reactive radicals that can convert almost any organic substance in carbon dioxide. The AOPs family includes different processes such as the Fenton reaction, ozonation, semiconductor photocatalysis, UV/H_2_O_2_, electrochemical oxidation, sonochemistry, wet air oxidation, activated persulfate, and others [9].

Among AOPs, electrochemical oxidation stands out for its ease of use, outstanding performance, and the ability to combine with renewable energy sources, thus providing an integrated green solution [10]. In recent decades, the study of anodic oxidation for environmental remediation has become more and more intense, due to the emergence of new electrode materials. Among them stands boron-doped diamond (BDD), with exciting properties, such as excellent stability and high potential window [11].

Like most heterogeneous AOPs, the efficiency of electrochemical oxidation is mainly limited by two restrictions [12].

(i) Mass transport at the electrode surface where most of the reactions occur.
(ii) The concentration of reactive oxygen species.

An exciting alternative to cope with both problems is the use of sonochemistry. It is well known that the use of ultrasound can increase mass transfer [13]. Besides, the use of the low-medium frequency of ultrasound waves has physicochemical effects in aqueous solution, due to a phenomenon called acoustic cavitation [14]. Briefly, under the influence of high-power ultrasound waves, cavities that are expanded and crashed adiabatically are formed. According to the “hotspot theory,” the conditions (i.e., temperature and pressure) inside these bubbles are very extreme. Therefore, the pyrolysis of volatile species inside or near the interface of the cavity can occur. At the same time, reactive oxygen species are produced inside the cavity and at the interface with the solution due to water cleavage. These radicals are released when the cavity cannot expand anymore and collapses internally [15,16].

Nowadays, there are several reports for the combination of different electrodes, like dimensionally stable anodes (DSA), as well as with non-active materials, like the boron-doped diamond with ultrasonic radiation.

Tran et al. [17] examined the synergistic action of ultrasound at 520 kHz and electrochemical oxidation using Ti/PbO_2_ as the anode at 1–15 A for the degradation of carbamazepine. They found that the synergistic action was higher at the low current density. The synergy was 33% for ultrasound power 40 W and a current equal to 1 A. Bringas and coworkers [18] investigated the combination of BDD mediated anodic oxidation and low-frequency ultrasound (20 kHz) for the sonoelectrolysis of the herbicide diuron. After 8 h, the rate constant for the mineralization of 26 mg/L diuron was 0.015, 0.156, and 0.305 h^−1^ for ultrasound, electrooxidation at 60 mA/cm^2^, and 10 g/L Na_2_SO_4_ and the hybrid process, respectively. Thus, the estimated synergy between the two processes was 43%. Zhao et al. [19] studied the synergy of electrochemistry and ultrasound using BDD or Pt electrode at 20 mA/cm^2^ and 50 W of ultrasound at 33 kHz for the oxidation of phenol (240 mg/L) at acidic conditions (pH 3) and 0.05 M Na_2_SO_4_. They found that the apparent kinetic constant using BDD increased from 6.46 × 10^−5^ for electrochemical oxidation, to 2.59 × 10^−4^ s^−1^ with the simultaneous use of ultrasound.

In a follow up of this work, Zhao et al. [20] examined the sonoelectrochemical decomposition of phthalic acid over a BDD. They found that the mass transport coefficient increased by 199% for the hybrid process under the same experimental conditions. Ren et al. [21] studied the hybrid process for the destruction of triclosan. The researchers used high-frequency ultrasound (850 kHz) with stainless steel as both anode and cathode. Under optimized conditions, the researcher achieved an 82% degradation of 5 mg/L of Triclosan in 15 min, while the apparent kinetic constant was 0.115 min^−1^. Souza et al. [22] investigated the sonoelectrolysis of dimethyl phthalate (DMP) with BDD and low-frequency ultrasound operated at 24 kHz. The authors conclude that the presence of ultrasound did not significantly improve DMP degradation. On the other hand, the mineralization was enhanced with sonoelectrochemical oxidation.

Recently, Dietrich and coworkers [23] investigated the combination of BDD electrodes with acoustic cavitation at 24 kHz for the destruction of the endocrine disruptor Bisphenol A. The researchers calculated a synergistic ratio up to 220% when the electrodes were operated at 5 V. However, the synergistic action disappeared when the potential increased to 10 V. The synergy index was limited to ca 1. Very recently, Dionisio et al. [24] studied the decomposition of methyl paraben by the combined action of electrochemical oxidation and ultrasound. The authors tested different ultrasonic frequencies (20 kHz, 1, and 10 MHz) and focused on the effect of chlorine and sulfate ions. They found that low-frequency ultrasound showed greater efficiency due to the activation of electrogenerated persulfate and increased the production of oxidizing species.

Although there is sufficient literature on hybrid processes such as sonoelectrochemical oxidation, a significant problem is how the synergies of the two processes are examined. In most cases, the first process is studied in depth (optimized), and then the role of the second is examined in the specific experimental conditions, that is, in a momentary representation of the operation of the system (snapshot). There is a risk that the results of the system behavior will be generalized (extrapolated), which may lead to incorrect conclusions. It is, therefore, imperative that both methods be studied in a broader range of operating parameters. Furthermore, to the best of our knowledge, the literature usually focuses on the effect of electrolyte and has not discussed the role of the water matrices in the observed synergistic action between ultrasound and electrochemical oxidation in environmentally relevant conditions. Since the applications of the AOPs are targeted in real matrices, it is crucial to study the efficiency of the hybrid processes in complex systems. Therefore, this work aims to cover this gap and to explore the synergistic sonoelectrooxidation of parabens, with an emphasis on the role of the water matrix on the observed efficiency and synergy, in comparison with the separated processes.

## 2. Materials and Methods

### 2.1. Chemicals

Propyl Paraben (>99% purity), humic acid, sodium chloride, and sodium bicarbonate were supplied from Aldrich. Sodium sulfate was purchased from Scharlau. Acetonitrile HPLC grade was supplied from PanReac AppliChem (Barcelona, Spain). The physico-chemical characterization of the synthetic or environmental matrices investigated is summarized in Table 1.

### 2.2. Experimental

The reactor used for both anodic oxidation and sonolysis was cylindrical, made of glass, and double wall with volume 200 mL. BDD (supplied by Adamant Technologies ((La Chaux-de-Fonds, Switzerland)) with 8 cm^2^ of surface area was served as the anode, and a Titanium sheet 8 cm^2^ was the cathode. Electricity was provided using a PeakTech 1885 programmable laboratory power supply. Almost all experiments were performed using 0.1 M Na_2_SO_4_; a relatively inert electrolyte, except when otherwise noted. The reactor was connected with a Grant LVF6 water bath to control the temperature at 25 °C.

Sonochemical and sono-electrochemical experiments were conducted in the same reactor, while the apparatus was used as described elsewhere [25]. Briefly, the ultrasound generator used was a Branson sonifier 450 (450 W), operated at 20 kHz. The transmitter was a horn made from titanium with 1 cm^2^ diameter and was placed at the center between the two electrodes. The ultrasound power inside the reactor was determined calorimetrically, according to the work of Kimura et al. [26] All experiments were performed twice, and the difference between the experimental measurements did not exceed 5%, while the duration of most experiments was 30 min. Table 2 summarizes the experimental conditions, the observed kinetic constants, the half-life and the percentage removal of PP after 10 min of treatment.

### 2.3. High-Performance Liquid Chromatography

The analysis of propyl paraben was based on previous work [27]. In brief, the propyl paraben was monitored using a Waters Alliance 2695 liquid chromatography system. The separation was achieved by a Kinetex (Phenomenex, Torrance, CA, USA) C18 2.6 μm 150 × 2.1 mm column. An isocratic elution with a mixture of 25:75 water: acetonitrile was used, and the flow rate was 0.25 min^−1^. Propyl paraben was detected using a PDA detector at 254 nm (Waters 2996).

### 2.4. Total Organic Carbon and UV_254_

An AURORA 1030 W analyzer (OI Analytical, College Station, TX, USA) was used for the measurement of total organic carbon (TOC). The absorbance at 254 nm (UV_254_) was measured using a HACH (Dr. Lange) DR5000 spectrophotometer and quartz cuvettes.

### 2.5. Calculation of Synergy

Assuming that both processes follow pseudo-first-order kinetics, the Synergy (S) can be calculated from Equation (1) [28]:

(1) $$S=1-\frac{{(k}_{\mathrm{AO}}{+k}_{\mathrm{US}})}{k_{\mathrm{AO}/\mathrm{US}}}\times 100\;(\%)$$

where k_AO/US_, k_AO_, and k_US_ are the apparent kinetic constants for sonoelectrochemical, electrochemical anodic oxidation, and sonolysis.

(2) $$\mathrm{where}\;S=\left\{\begin{array}{l} >\;0\;\mathrm{synergistic}\;\mathrm{effect}\\ =0\;\mathrm{cumulative}\;\mathrm{effect}\\ <\;0\;\mathrm{antagonistic}\;\mathrm{effect} \end{array}\right\}$$

## 3. Results and Discussion

### 3.1. Effect of Current and Ultrasound Power

**Figure 1:**
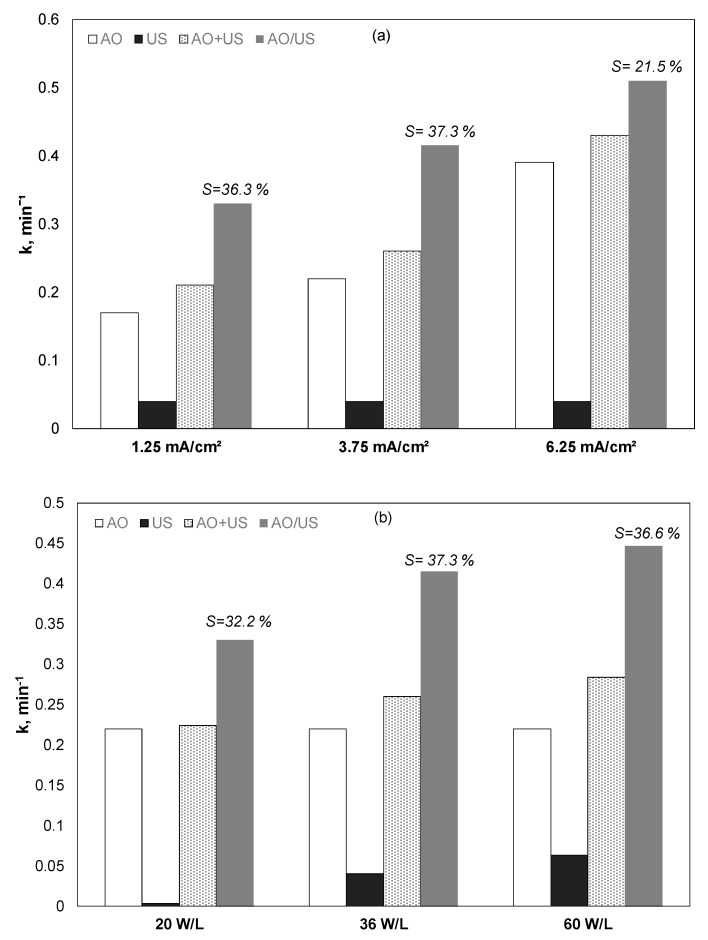
(Apparent) kinetic constant for the removal of 0.5 mg/L propyl paraben by anodic oxidation (AO), ultrasound (US), theoretical sum (AO + US), and combined (AO/US) process. (**a**) Effect of current density, US = 36 W/L; (**b**) effect of ultrasound power. Conditions: [Na_2_SO_4_] = 0.1 M, 3.75 mA/cm^2^, and inherent pH.

Initially, the effect of the current was examined in the presence or not of 36 W/L of ultrasound at the frequency of 20 kHz, and the results are shown in Figure 1a and Table 2. k_AO_, k_US_, K_AO+US_, K_AO/US_ denotes the apparent kinetic constant for electrochemical oxidation, ultrasound, the theoretical sum of electrochemical oxidation plus ultrasound, and the combined (simultaneous) use of electrochemical oxidation and ultrasound, respectively.

In both cases, increasing the current increased the PP removal rate. The observed kinetic constant for electrochemical oxidation alone increased from 0.17 to 0.39 min^−1^ when the current increased from 1.25 to 6.25 mA/cm^2^. At the same time, the observed kinetic constant for the combined (EO/US) process increased from 0.33 to 0.51 min^−1^.

According to Comninellis et al. [12], increasing the current increased the reactive oxygen species (Equation (3)), up to a point where oxygen evolution occurs

(3) $$BDD\to \left(H_{2}O\right)\to BDD\left({}^{•}OH\right)+H^{+}+e^{-}$$

Applying Equation (2) to the data of Figure 1a and Table 2 shows that the percentage of synergy decreases from 36.3% to 21.5% when the current intensity increases from 1.25 to 6.25 mA/cm^2^. In other words, for a (relatively) high concentration of reactive oxygen species, the role of ultrasound to produce additional (in comparison with electrochemical oxidation) oxidative species is not as crucial as at low current densities.

Additional experiments were performed using a constant current at a relatively low applied current of 3.75 mA/cm^2,^ while the sonochemical power density changed from 20 to 60 W/L (Figure 1b). Despite that increase, the intensity of ultrasound has resulted in the increased removal of propyl paraben; the degree of synergism appears to exhibit a maximum at 36 W/L. Nevertheless, for any combination of current density and ultrasound power, the simultaneous use of the two processes (AO/US) was higher than the theoretical sum (AO + US), indicating a true synergy between electrochemistry and sonochemistry, at least at the conditions studied.

### 3.2. Effect of PP Initial Loading

**Figure 2:**
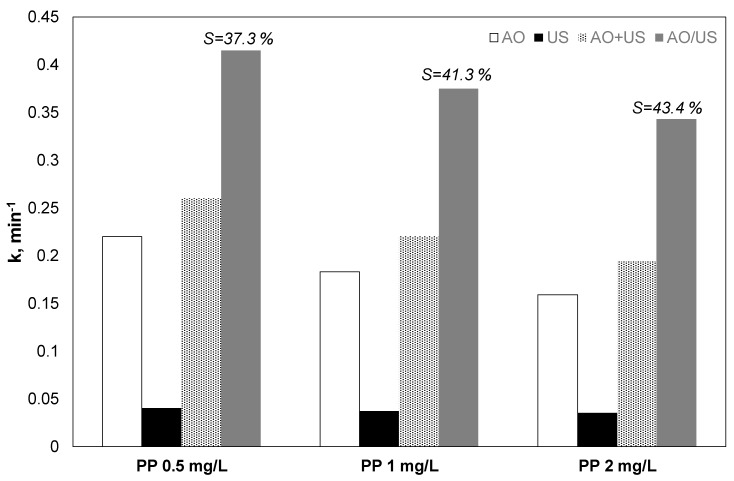
Effect of paraben initial concentration on the kinetic constant for the removal of propyl paraben by electrochemical anodic oxidation (AO), ultrasound (US), theoretical sum (AO + US) and combined (AO/US) process [Na_2_SO_4_] = 0.1 M, 3.75 mA/cm^2^, 36 W/L at 20 kHz and inherent pH.

The influence of propyl paraben initial loading on their oxidation with separated and combined processes was examined, and the results are shown in Figure 2. The observed constant for both hybrid and separated processes decreased when the concentration of the organic increased. However, the observed trend of reduction was less for the combined process.

Assuming that the degradation of parabens was mainly due to the hydroxyl radicals and pseudo-first-order kinetics, the propyl paraben destruction can be described from the following equation:

(4) $$\left[\mathrm{PP}\right]\overset{\mathrm{EO}}{\underset{\mathrm{US}}{\to }}k_{\mathrm{PP},\mathrm{OH}}\left[\mathrm{PP}\right][{}^{•}\mathrm{OH}{]}_{\mathrm{SS}}$$

The steady-state concentration of hydroxyl radicals depends on the operating parameters (i.e., sonochemical power, frequency, and pH for ultrasound) or (current density, electrolyte, and pH for electrochemical oxidation). It can be considered constant for given experimental conditions. Therefore, Equation (5) can be simplified as follows:

(5) $$\left[\mathrm{PP}\right]\overset{\mathrm{EO}}{\underset{\mathrm{US}}{\to }}k_{app}\left[\mathrm{PP}\right]$$

Thus, for a high ratio of organics/reactive oxygen species, the concentration of ROS becomes the limiting factor. Since the synergistic action of ultrasound and electrochemistry leads to a higher concentration of ROS, this ratio for the hybrid process is lower.

Indeed, in the pioneering work of Kapalka et al. [29], a kinetic model for the electrooxidation of the organic pollutants was proposed. Organics removal is divided into two distinct regions. Chemical oxygen demand (COD) decreased linearly with treatment time when the process was under current control, while the COD decreased exponentially with the treatment time when mass transport controls the efficiency. There are several reports about the ultrasound-induced degradation of different organics where the pollutant conversion follows pseudo-first-order kinetics. Frontistis et al. [30] tested the sono-degradation of 17a ethinylestradiol (EE2) using a sonicator operated at 80 kHz. The abatement of EE2 followed the first-order reaction rate when the EE2 loading was between 25–110 μg/L. However, with a further increase in EE2 concentration, a transition of the reaction kinetics to a lower order was observed.

Despite that, when the propyl paraben loading raised from 0.5 to 2 mg/the apparent constant declined from 0.413 to 0.343 min^−1^, and the calculated synergy percentage raised from 37.3% to 43.4%, making the use of the hybrid process more attractive for larger initial concentrations of pollutants.

### 3.3. Effect of pH

**Figure 3:**
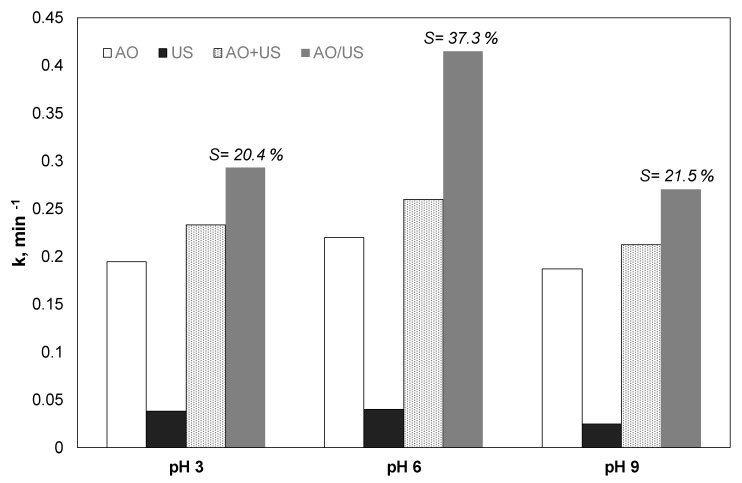
Effect of pH on the kinetic constant for the degradation of 0.5 mg/L paraben by electrochemical anodic oxidation (AO), ultrasound (US), theoretical sum (AO + US) and combined (AO/US) process [Na_2_SO_4_] = 0.1 M, 3.75 mA/cm^2^, 36 W/L at 20 kHz.

Another group of experiments was performed in alkaline (9), acidic (3), and neutral (6) pH, to explore the effect of the pH in the combined and the separate processes. The results are depicted in Figure 3.

Sonolysis was slightly favored in neutral pH. The apparent constant was reduced from 0.0401 to 0.025 min^−1^ for pH 6 and 9, respectively. Anodic oxidation also showed marginally better removal at neutral conditions; however, the differences between the observed kinetic constants were less than 15% for the condition in question.

The pH changes can influence, in two distinct ways, the efficiency of AOPs studied in this work:

(i) Changing the electrostatic forces between the pollutant and the anode in the case of electrochemical oxidation or the micro-bubbles formed in the presence of the ultrasonic field. Propyl paraben has a pKa of 8.4. Therefore, in alkaline conditions, it is negatively charged. Thus, a possible explanation for the reduced efficiency of sonochemical and sonoelectrochemical degradation in alkaline conditions is the electrostatic repulsions between the propyl paraben and the negatively charged liquid-bubble interface.
(ii) The change in pH significantly affects the generation of reactive oxygen species.

For electrochemical oxidation, according to Murugananthan et al. [31], the production of hydroxyl radicals is favored in alkaline pH:

(6) $${\mathrm{OH}}^{-}\overset{\mathrm{EO}}{\to }{}^{•}{\mathrm{OH}}_{\left(\mathrm{ads}\right)}+e^{-}$$

Wu et al. [32] measured the effect of the pH on the sono generation of hydroxyl radicals at 20 kHz, using p-CBA as the probe compound. They found that the concentration of hydroxyl radicals fluctuates between 0.05 at pH 8 and 0.01 at pH 7.

### 3.4. Effect of Chloride Ions

**Figure 4:**
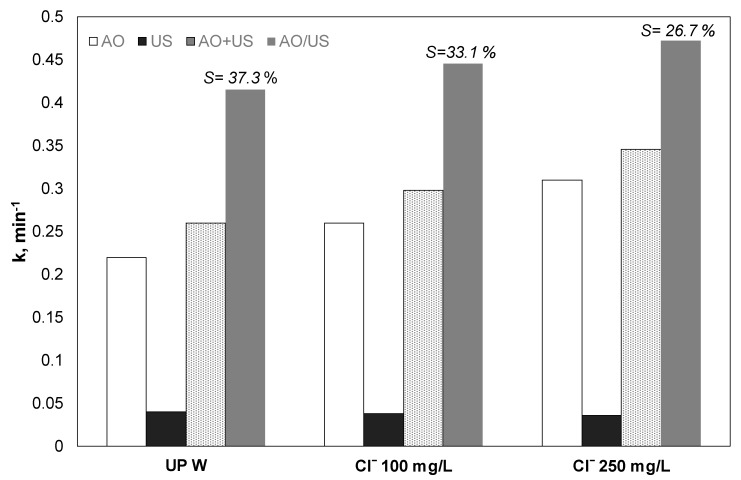
Effect of chlorides on the kinetic constant for the removal of 0.5 mg/L propyl paraben by electrochemical anodic oxidation (AO), ultrasound (US), theoretical sum (AO + US), and combined (AO/US) process. Conditions: [Na_2_SO_4_] = 0.1 M, 3.75 mA/cm^2^, 36 W/L at 20 kHz and inherent pH.UPW: Ultrapure water.

The role of chloride ions in the observed synergy and the separated processes was also investigated. Experiments were conducted with chloride ions up to 250 mg/L. As shown in Figure 4, the existence of chloride increases PP removal. However, the degree of synergy decreased from 37.3% to 26.7%. To examine the role of chloride in the separate and the combined process, additional experiments, with only sonolysis and electrochemical oxidation with 100 and 250 mg/L Cl^−^, were conducted. As shown in Figure 4, the presence of chlorides showed controversial results for the two processes. Analytically the presence of chlorides increased the apparent constant significantly for electrooxidation, where the rate constant increased from 0.22 to 0.26 and 0.31 min^−1^ for ultrapure 100 and 250 mg/L Cl^−^, respectively. Meanwhile, the corresponding values for the ultrasound case slightly decreased from 0.0401 to 0.038 and 0.036 min^−1^.

Bosio et al. [33] tested the electrooxidation of a mixture of parabens (10 mg/L each) using the Ti/Pt anode. The researchers observed a 100% removal of all parabens after 10 min using 3 g/L NaCl and a 75 A/m^2^ applied current. They found that the efficiency was much more significant when sodium chloride was used as an electrolyte instead of sodium sulfate. At the same time, the further increase in the concentration of NaCl from 3, which was optimal to 5 g/L, did not reduce the required treatment time. The authors attributed the observed results to the fact that the reactive sulfate species were less active than the reactive chlorine species for the degradation of parabens.

It is well known that the existence of chloride induced the electro-production of active chlorine species according to the reactions [34]:

(7) $${2\mathrm{Cl}}^{-}\to {\mathrm{Cl}}_{2}{+2e}^{-}$$

(8) $${\mathrm{Cl}}^{-}\to {\mathrm{Cl}}^{•}+e^{-}$$

(9) $${\mathrm{Cl}}^{•}+{\mathrm{Cl}}^{•}\to {\mathrm{Cl}}_{2}$$

(10) $${\mathrm{Cl}}^{•}{+O}_{2}\to {\mathrm{ClO}}_{2}$$

(11) $${\mathrm{Cl}}_{2(\mathrm{aq})}{+\mathrm{OH}}^{-}\to {\mathrm{ClO}}^{-}{+\mathrm{Cl}}^{-}{+H}_{2}O$$

(12) $$\mathrm{HClO}⇄{\mathrm{ClO}}^{-}{+H}^{+}$$

Since the active chlorine has a longer lifespan than the hydroxyl radicals, they are easier to diffuse into the bulk solution, “bypassing” the mass transfer limitations occurring at the electrode surface for electrochemical oxidation, via hydroxyl radicals or direct electron transfer. Therefore, the apparent increase of mass transfer in the case of ultrasound is probably not as necessary as in the case of ultrapure water, where the oxidation was limited at or near the electrode surface. Also, the propyl paraben is not volatile to diffuse inside the ultrasound-induced bubble, where pyrolytic decomposition can occur, according to the “hot spot” theory.

### 3.5. Effect of Bicarbonates

**Figure 5:**
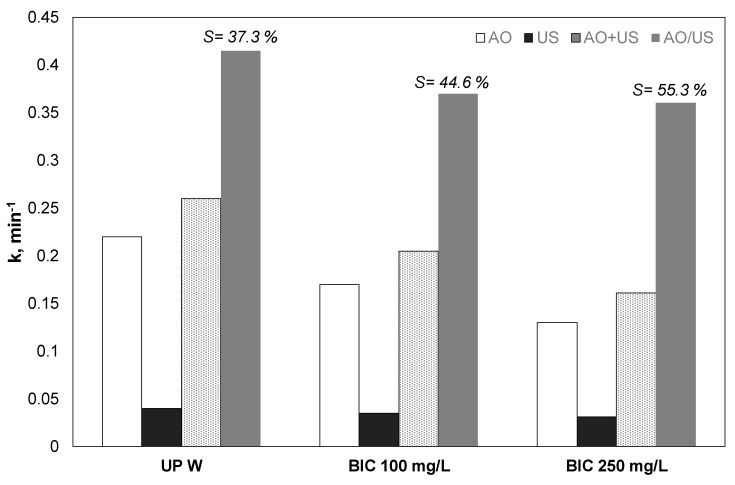
Effect of humic acid on the kinetic constant for the removal of 0.5 mg/L propyl paraben by electrochemical anodic oxidation (AO), ultrasound (US), theoretical sum (AO + US) and combined (EO/US) process [Na_2_SO_4_] = 0.1 M, 3.75 mA/cm^2^, 36 W/L at 20 kHz and inherent pH. UPW: Ultrapure water and 0.1 M Na_2_SO_4_, HA: Humic acid.

Since different environmental matrices contain carbonates and bicarbonates, their effect was assessed in another group of experiments, and the apparent kinetic constants are shown in Figure 5. Bicarbonate decreased the efficiency of both electrooxidation and sonolysis. However, the decrease observed in the case of ultrasound was significantly lower. For the sonodegradation and electrochemical degradation of 500 μg/L of PP, the kinetic constant was reduced by 56% and 34%, respectively.

However, this decrease was lower for the hybrid process.

Usually, carbonates and bicarbonates act as radical scavengers, thus lowering the efficiency of AOPs and their applications in real or industrial wastewater according to the reactions [35,36]:

(13) $${}^{•}\mathrm{OH}+{\mathrm{CO}}_{3}^{2-}\overset{k=3.9\;\times \;10^{8}\;{\mathrm{mol}}^{-}{}^{1}{\mathrm{dm}}^{3}s^{-}{}^{1}}{\to }{\mathrm{CO}}_{3}^{•-}+{\mathrm{OH}}^{-}$$

(14) $${}^{•}\mathrm{OH}+{\mathrm{HCO}}_{3}^{-}\overset{k=8.5\;\times \;10^{6}\;{\mathrm{mol}}^{-1}{\mathrm{dm}}^{3}s^{-1}}{\to }H_{2}O+{\mathrm{CO}}_{3}^{•-}$$

Nevertheless, depending on the target pollutant, there are reports that the presence of carbonates is increasing the process efficiency. Despite that, in general, carbonate radicals are considered to have less oxidizing power than hydroxyl radicals; they are active for longer. Hence, if the target pollutant is an electron-rich organic compound with a kinetic constant (k) for the reaction with carbonate radical greater than 10^8^ M^−1^ s ^−1^ like, for example, sulfonamides, it is expected that the presence of carbonates will improve the removal rate [37].

### 3.6. Effect of Natural Organic Matter (Humic Acid)

**Figure 6:**
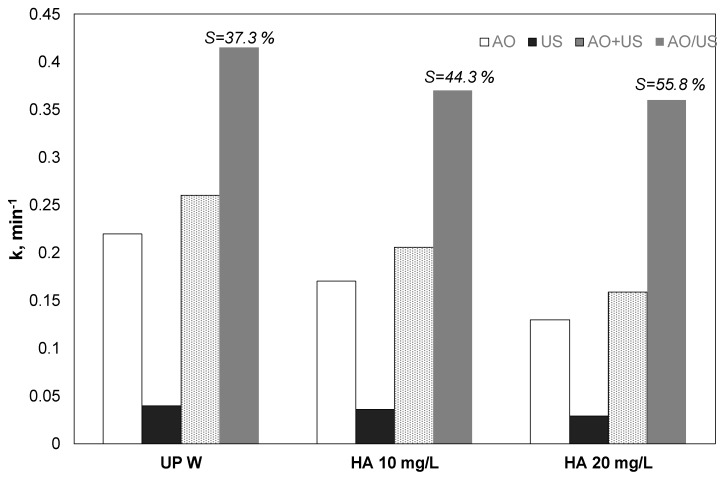
Effect of humic acid on the kinetic constant for the removal of 0.5 mg/L propyl paraben by electrochemical anodic oxidation (AO), ultrasound (US), theoretical sum (AO + US) and combined (EO/US) process [Na_2_SO_4_] = 0.1 M, 3.75 mA/cm^2^, 36 W/L at 20 kHz and inherent pH. UPW: Ultrapure water and 0.1 M Na_2_SO_4_, HA: Humic acid.

Environmentally relevant matrices like surface water or secondary effluents contain organic matter that is usually resistant to further oxidation [38]. Humic acid in concentrations up to 20 mg/L was used to simulate the presence of organics in environmental samples. The results for the separated and the combined process and the estimated theoretical sum were depicted in Figure 6.

PP removal in the existence of HA decreased for both anodic oxidation and sonolysis; the synergy was notably enhanced from 37.3% to 55.8%.

There are several reports in the literature referring to the role of humic acid and natural organic matter or organic loading on the efficiency of electrochemical oxidation and ultrasound.

Our experiments corroborate the work of Zhou et al. [19], who tested the electrochemical destruction of microcystin LR over BDD or the IrO_2_Ta_2_O_5_/Ti anode. The existence of 10 mg/L natural organic matter at 5 mA/cm^2^ decreased the observed kinetics for the elimination of 0.2 μM microcystin almost 2.3 times from 0.337 to 0.149 min^−1^.

Latterly, Lianou et al. [39] investigated the sonodegradation of the anti-inflammatory drug Piroxicam, using a 20 kHz ultrasound. The researchers observed a remarkable delay when the HA concentration increased. The observed constant for the destruction of 320 μg/L using 36 W/L of sonochemical power decreased almost three times from 0.21 to 0.071 min^−1^ for pure water and 10 mg/L HA [39].

Therefore, in the hybrid EO/US system, there is a competition between the humic acid and the pollutant for the reaction with the radicals, the adsorption at the electrode surface, and the accumulation at the bubble interface or the diffusion inside the cavity. 10 mg/L of HA has 4.2 mg/L of carbon, that is almost 12.5 times higher than the TOC contained on 500 μg/L of propyl paraben. Therefore, assuming that the ROS attack all the organic molecules with similar reactivity (rate constants ≈ 10^8^ M^−1^ s^−1^), the chance for a radical to react with the paraben is almost 8% in comparison with the humic acid.

### 3.7. Effect of Water Matrix

**Figure 7:**
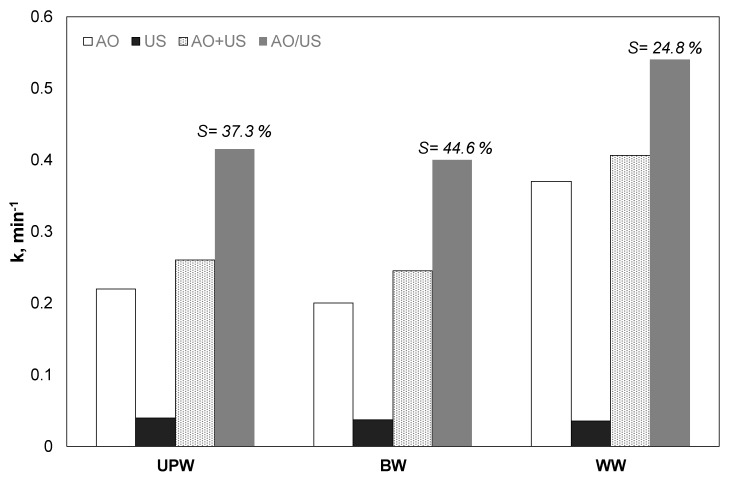
(Apparent) kinetic constant for the removal of 0.5 mg/L propyl paraben by electrochemical anodic oxidation (AO), ultrasound (US), theoretical sum (AO + US) and combined (AO/US) process in different water matrices [Na_2_SO_4_] = 0.1 M, 3.75 mA/cm^2^, 36 W/L at 20 kHz and inherent pH.

Probably the most interesting part about studying the coupling of different processes is what happens to real wastewater or environmental samples. The study of the use of technologies such as advanced oxidation processes in actual/complex water matrices has increased significantly in recent years [40]. Although in the past, they were usually associated with significantly reduced performance, newer studies show that this is not always the case [40,41]. Under this perspective, additional tests were performed to assess the impact of the water matrix on the synergistic action of the ultrasound and the electrochemical oxidation with different environmentally relevant water matrices: Ultrapure water (UP), bottled water (BW) and wastewater effluent (WW). As shown in Figure 7, while bottled water and ultrapure water showed similar efficiency, the degree of synergy was decreased in the case of wastewater. A possible explanation involves the role of chloride, as discussed already in Section 3.6. For the secondary effluent, there is a remarkable increase in efficiency with indirect electrochemical oxidation, due to the presence of chloride.

It is worth mentioning that there are conflicting results in the literature for the electrochemical oxidation of different micropollutants in secondary effluents [42,43]. For example, the electrochemical oxidation of 100 μg/L of EE2 on BDD was favored in the secondary effluent compared to pure water [42]. In contrast, in another study from the same research group, the observed kinetic constant for the removal of pesticide thiamethoxam in secondary effluent was reduced by about eleven times compared to ultrapure water [43]. These results imply that the performance of electrochemical oxidation depends on the nature of the pollutant, the composition of the water matrix, and the operating parameters. Another possible explanation for the different behavior of the water matrices in electrochemical oxidation with other advanced oxidation processes is the formation of an acidic diffusion boundary layer during the anodic oxidation of water. This acidic layer can lower the scavenging effect of anions like bicarbonates significantly [44].

On the other hand, the efficiency decreased in all processes due to the existence of the remaining ions and the organic matter. An interesting observation is that for sonodegradation, the rate constant was only marginally decreased from 0.041 to 0.037 and 0.036 min^−1^ for UP, BW, and WW, respectively. The results are in agreement with another study of our group [30] that examined the sonochemical oxidation of 17α-ethynylestradiol at 80 kHz and 46 W/L. The degradation of EE2 was only slightly decreased when secondary effluent was used instead of ultrapure water. Indeed, in another work [25], where the researchers studied the sonodegradation of ethyl paraben, the researchers concluded, with the help of factorial design methodology, that the impact of the water matrices was not statistically significant, at least not at the range of the parameters studied.

Interestingly, the synergistic action was enhanced when experiments were conducted on bottled instead of ultrapure water. The synergy S increased from 37.3% in ultrapure water to 44.6% for bottled water. This increase can be related to the presence of bicarbonates, since, as shown in Table 1, the bottled water contains ca 200 mg/L BIC. Interestingly, the rate constant for the combined constant was almost identical for BW and UP (ca 0.4 min^−1^). Overall, the results related to different aqueous matrices are in agreement with the results obtained with the different ions (i.e., bicarbonates and chlorides) and the presence of natural organic matter.

To provide a more comprehensive study of the synergy between the two processes, the elimination of total organic carbon (TOC) and the absorbance at 254 nm in the case of secondary effluent were also studied. The TOC removal after 15 min of treatment was 19%, 6%, and 36% for the anodic oxidation, the ultrasound irradiation, and the hybrid process, respectively. For the same treatment time, the UV_254_ decreased by 30%, 13%, and 54%, respectively. These results show that synergy is not limited to paraben. There is a synergy in both the oxidation of persistent organic matter of the WW and the by-products of oxidation.

Indeed, Dionisio et al. [24] have shown that the simultaneous use of low-frequency ultrasound and oxidation on boron-doped diamond significantly accelerated the decomposition of intermediate products. At the same time, the researchers observed that the production of perchlorate began after the complete mineralization. The researchers suggested that synergy due to ultrasound in complex water matrices is due to both natural and chemical phenomena. In addition to the well-known improvement in mass transport, many different oxidizing species coexist in the hybrid process reactor. The result of this coexistence within the sound field, along with their different lifespan and the selectivity of oxidants between the different substances and ions present in the solution, can significantly alter the performance of the hybrid process.

## 4. Conclusions

The synergistic action of electrochemical oxidation and sonochemistry with emphasis on the impact of the water matrices and operating conditions was studied. This work, excluding cost analysis, highlights the need to examine the efficacy of hybrid processes (as well as of individual AOPs), in actual water matrices, instead of ultrapure water and synthetic solutions. The main conclusions derived from this work are:

- In most cases, the simultaneous application of the two processes showed a significant synergistic effect. However, the degree of synergy was strongly dependent on the conditions in question;
- In general, the synergy degree was increased in the existence of humic acid, bicarbonates, and bottled water;
- Synergy was decreased in the presence of chloride and WW, possibly due to the crucial role of active chlorine species induced by electrochemical oxidation. Therefore, in terms of degradation efficiency, the simultaneous use of ultrasound is not crucial for complex wastewater that contains significant amounts of chlorides. The above results are the first step to a more comprehensive study of hybrid processes in different aqueous matrices, in a large window of operating parameters that will include both toxicity and cost estimation in future work.

## Figures and Tables

**Table 1:** Physico-chemical characterization of different water matrices.

Property	Ultrapure Water (UPW) (+ 0.1 M Na_2_SO_4_)	Bottled Water (BW) (+ 0.1 M Na_2_SO_4_)	Wastewater Effluent (WW) (+ 0.1 M Na_2_SO_4_)
pH	6.3	7.2	8.1
Conductivity, mS/cm	16.3	16.7	17.2
TOC, mg/L			7.7
Bicarbonate, mg/L		198	217
Chloride, mg/L		7.4	68
Nitrate, mg/L		2.1	4.7

**Table 2:** Summarized data from Figure 1, Figure 2, Figure 3, Figure 4, Figure 5, Figure 6 and Figure 7. HA: Humic Acid. BIC: Bicarbonates, BW: Bottled Water, WW: Wastewater.

[PP]_0_ (mg/L)	Current Density (mA/cm^2^)	US Power (W/L)	Water Matrix	pH	k_app_ × 10^−3^ min^−1^	t_1/2_ (min)	Removal (10 min)
0.5	1.25		0.1 M Na_2_SO_4_	inherent	170	4.1	87%
0.5	3.75		0.1 M Na_2_SO_4_	Inherent	220	3.2	89%
0.5	6.25		0.1 M Na_2_SO_4_	Inherent	390	1.8	98%
0.5		36	0.1 M Na_2_SO_4_	inherent	40	17.3	32%
0.5	1.25	36	0.1 M Na_2_SO_4_	Inherent	330	2.1	95%
0.5	3.75	36	0.1 M Na_2_SO_4_	Inherent	410	1.7	98%
0.5	6.25	36	0.1 M Na_2_SO_4_	Inherent	510	1.4	99%
0.5		20	0.1 M Na_2_SO_4_	Inherent	4	173.3	3%
0.5		60	0.1 M Na_2_SO_4_	inherent	63	11.0	47%
0.5	3.75	20	0.1 M Na_2_SO_4_	Inherent	330	2.1	97%
0.5	3.75	60	0.1 M Na_2_SO_4_	Inherent	447	1.6	99%
1	3.75		0.1 M Na_2_SO_4_	Inherent	183	3.8	82%
2	3.75		0.1 M Na_2_SO_4_	Inherent	159	4.4	81%
1		36	0.1 M Na_2_SO_4_	Inherent	37	18.7	29%
2		36	0.1 M Na_2_SO_4_	Inherent	35	19.8	32%
1	3.75	36	0.1 M Na_2_SO_4_	Inherent	375	1.8	98%
2	3.75	36	0.1 M Na_2_SO_4_	Inherent	343	2.0	97%
0.5	3.75		0.1 M Na_2_SO_4_	3	195	3.6	84%
0.5		36	0.1 M Na_2_SO_4_	3	38	18.2	31%
0.5	3.75	36	0.1 M Na_2_SO_4_	3	293	2.4	93%
0.5	3.75		0.1 M Na_2_SO_4_	9	187	3.7	83%
0.5		36	0.1 M Na_2_SO_4_	9	25	27.7	21%
0.5	3.75	36		9	270	2.6	94%
0.5	3.75		0.1 M Na_2_SO_4_ 100 mg/L Cl^−^	inherent	260	2.7	92%
0.5		36	0.1 M Na_2_SO_4_ 100 mg/L Cl^−^	inherent	38	18.2	33%
0.5	3.75	36	0.1 M Na_2_SO_4_ 100 mg/L Cl^−^	inherent	445	1.6	99%
0.5	3.75		0.1 M Na_2_SO_4_ 250 mg/L Cl^−^	inherent	310	2.2	95%
0.5		36	0.1 M Na_2_SO_4_ 250 mg/L Cl^−^	inherent	36	19.3	33%
0.5	3.75	36	0.1 M Na_2_SO_4_ 250 mg/L Cl^−^	inherent	472	1.5	99%
0.5	3.75		0.1 M Na_2_SO_4_ 100 mg/L BIC	inherent	170	4.1	84%
0.5		36	0.1 M Na_2_SO_4_ 100 mg/L BIC	inherent	35	19.8	33%
0.5	3.75	36	0.1 M Na_2_SO_4_ 100 mg/L BIC	inherent	370	1.9	98%
0.5	3.75		0.1 M Na_2_SO_4_ 250 mg/L BIC	inherent	130	5.3	75%
0.5		36	0.1 M Na_2_SO_4_ 250 mg/L BIC	inherent	31	22.4	26%
0.5	3.75	36	0.1 M Na_2_SO_4_ 250 mg/L BIC	inherent	360	1.9	97%
0.5	3.75		0.1 M Na_2_SO_4_ 10 mg/L HA	inherent	170	4.1	81%
0.5		36	0.1 M Na_2_SO_4_ 10 mg/L HA	inherent	36	19.3	32%
0.5	3.75	36	0.1 M Na_2_SO_4_ 10 mg/L HA	inherent	370	1.9	98%
0.5	3.75		0.1 M Na_2_SO_4_ 20 mg/L HA	inherent	130	5.3	74%
0.5		36	0.1 M Na_2_SO_4_ 20 mg/L HA	inherent	29	23.9	23%
0.5	3.75	36	0.1 M Na_2_SO_4_ 20 mg/L HA	inherent	360	1.9	97%
0.5	3.75		0.1 M Na_2_SO_4_ BW	inherent	200	3.5	84%
0.5		36	0.1 M Na_2_SO_4_ BW	inherent	37	18.7	32%
0.5	3.75	36	0.1 M Na_2_SO_4_ BW	inherent	400	1.7	98%
0.5	3.75		0.1 M Na_2_SO_4_ WW	inherent	370	1.9	98%
0.5		36	0.1 M Na_2_SO_4_ WW	inherent	36	19.3	30%
0.5	3.75	36	0.1 M Na_2_SO_4_ WW	inherent	540	1.3	100%
